# A targeted antibody-based array reveals a serum protein signature as biomarker for adolescent idiopathic scoliosis patients

**DOI:** 10.1186/s12864-023-09624-7

**Published:** 2023-09-04

**Authors:** Zhenxuan Shao, Zengjie Zhang, Yiting Tu, Chongan Huang, Liang Chen, Anna Sun, Sunren Sheng, Xiaolei Zhang, Yan Wu

**Affiliations:** 1 Department of Orthopedic Surgery, the Second Affiliated Hospital, Zhejiang University School of Medicine, Hangzhou City, Zhejiang Province People’s Republic of China; 2 Orthopedics Research Institute of Zhejiang University, Hangzhou City, Zhejiang Province People’s Republic of China; 3 Key Laboratory of Motor System Disease Research and Precision Therapy of Zhejiang Province, Hangzhou City, Zhejiang Province People’s Republic of China; 4 Clinical Research Center of Motor System Disease of Zhejiang Province, Hangzhou City, Zhejiang Province People’s Republic of China; 5 Department of Orthopedics, The Second Affiliated Hospital and Yuying Children’s Hospital of Wenzhou Medical University, Wenzhou, China; 6 Key Laboratory of Orthopedics of Zhejiang Province, Wenzhou, China; 7 Department of Clinical Laboratory, Children’s Hospital, Zhejiang University School of Medicine, National Clinical Research Center For Child Health, Hangzhou, China

**Keywords:** Adolescent idiopathic scoliosis, Serum biomarkers, Antibody-based array, FAP, CD23

## Abstract

**Background:**

Evident adolescent idiopathic scoliosis (AIS) incurs high treatment costs, low quality of life, and many complications. Early screening of AIS is essential to avoid progressing to an evident stage. However, there is no valid serum biomarker for AIS for early screening.

**Methods:**

Antibody-based array is a large-scale study of proteins, which is expected to reveal a serum protein signature as biomarker for AIS. There are two segments of the research, including biomarkers screening and validation. In the biomarkers screening group, a total of 16 volunteers participated in this study, and we carried out differentially expressed proteins screening via protein array assay between No-AIS group and the AIS group, through which GeneSet enrichment analysis was performed. In the validation group with a total of 62 volunteers, the differentially expressed proteins from screening group were verified by Enzyme-Linked immunosorbent assay (ELISA), and then multiple regression analysis.

**Results:**

In our study, there were twenty-nine differentially expressed proteins in AIS, through Protein array assay and GeneSet enrichment analysis in the biomarkers screening group. Then the expression of FAP, CD23 and B2M decreased as the degree of AIS increased via ELISA in validation group (FAP, *p* < 0.0001; CD23, *p* = 0.0002; B2M, *p* < 0.0001). Further, the results of multiple regression analysis showed that FAP, CD23 are linked to Cobb angle, whereas B2M were excluded because of multicollinearity.

**Conclusions:**

Altogether, we found that serum protein FAP and CD23 are intimately related to AIS, suggesting FAP and CD23 are expected to serve as the serum biomarkers, which significantly facilitate frequent longitudinal monitoring as to keep track of disease progression and tailor treatment accordingly.

**Supplementary Information:**

The online version contains supplementary material available at 10.1186/s12864-023-09624-7.

## Background

Adolescent idiopathic scoliosis (AIS) is the most common form of spine deformity among adolescents [1], with an incidence is between 1 and 4%, which begins in early puberty [2, 3]. According to the Scoliosis Research Society, scoliosis is characterized as a lateral curvature of the spine that greater than 10 degrees as measured using the Cobb method on a standing radiograph [4]. Currently, AIS patients with early correction of spinal curvature have a good prognosis; instead, overlooking early screening, AIS patients with large curves had to face adverse long-term health outcomes in later adulthood, including an increased risk for shortness of breath with curves greater than 50°, diminished lung volumes with curves greater than 70°, and more impaired pulmonary function with curves greater than 100° [5].

Screening adolescents in the early stages of AIS development is the only meaningful secondary prevention strategy [6]. Currently, screening for AIS usually uses forward bending test (FBT), and the scoliometer measurement of angle of trunk rotation (ATR), which depends on subjective judgment and leads to high human and economic costs; the objective examination is based on an X-ray of the full length of spine, which is detrimental to the health of adolescence [3]. An objective, affordable and radiation-free method for early screening is urgently required.

Antibody-based array is a large-scale study of proteins, which is expected to reveal a serum protein signature as biomarkers for AIS. Importantly, finding biomarkers from biological samples just requires a common procedure without radiation hazard, which could significantly facilitate frequent longitudinal monitoring as to track disease progression closely and tailor treatment accordingly.

**Fig. 1:**
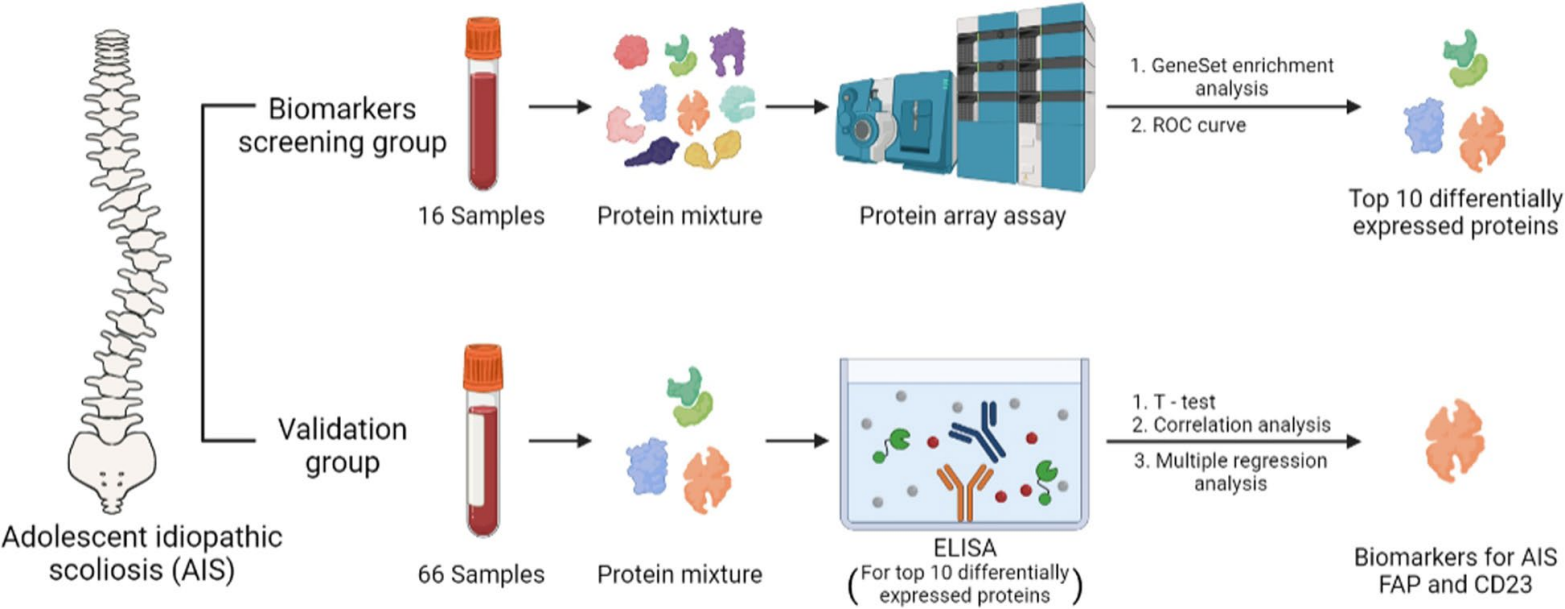
An overview of this study. There are two segments of the research, including biomarkers screening and validation. In the biomarkers screening group, a total of 16 volunteers were enrolled in this study, and there were ten differentially expressed proteins in AIS, through Protein array assay, GeneSet enrichment analysis and ROC curve. In the validation group, a total of 66 volunteers of different AIS degrades were recruited. Then, FAP, CD23 and B2M were underlined by the ELISA, and the expression of FAP, CD23and B2M decreased as the degree of AIS increased (FAP, *p* < 0.0001; CD23, *p* = 0.0002; B2M, *p* < 0.0001). Further, the results of multiple regression analysis and ROC curve showed that FAP, CD23 are linked to Cobb angle, but Age and B2M were excluded because of multicollinearity

In this study, we have used an antibody-based array to screen and quantify 640 proteins to identify potential serum biomarkers for AIS. As of writing, this paper is the first one to use an antibody-based array as a biomarker screening technique for AIS. There is not only a screening group, but also a validation group, in which we re-recruited 66 volunteers to validate differentially expressed proteins by Enzyme-Linked immunosorbent assay (ELISA) (Fig. 1 shows an overview of this study). Through above screening and verification, we found that FAP and CD23, between the No-AIS volunteers and the AIS patients, are intimately related to AIS, suggesting FAP and CD23 are expected to serve as the serum biomarkers.

## Results

### Demographic characteristics of study populations

In the biomarker screening group, the clinical features of the 16 patients were shown in Table 1. The volunteers were divided into the No-AIS group (*n* = 8) and the AIS group (*n* = 8); and in the AIS group, two volunteers were AIS-I (Cobb angle, 10°- 20°), three were AIS-II (Cobb angle, 20°- 40°), and three were AIS-III (Cobb angle, > 40°). As for the gender, the ratio of male to female was the same (3:5), and the age of two group was similar (No-AIS group, 11 ± 2.1 years; AIS group, 11 ± 3.5 years). BMI and Risser were similar in both groups (BMI, 19.9 ± 1.36 *vs* 19.8 ± 1.39; Risser, 2.0 ± 1.9 *vs* 2.0 ± 2.0, Table 1, Table S1).

**Table 1:** Demographic characteristics of study populations

	**Group**	**Cases (N)**	**Age (years) (mean + SD)**	**Gender (males/females)**	**BMI**	**Risser**
**Biomarker screening**	No-AIS (< 10°)	8	11 ± 2.1	3/5	19.9 ± 1.36	2.0 ± 1.9
	AIS (>10°)	8	11 ± 3.5	3/5	19.8 ± 1.39	2.0 ± 2.0
**Validation**	No-AIS (0°-10°)	10	12.2 ± 2.8	4/6	20.1 ± 2.13	2.0 ± 2.1
	AIS-I (10°-20°)	12	12.4 ± 2.4	7/5	19.6 ± 1.69	2.0 ± 1.9
	AIS-II (20°-40°)	16	14.2 ± 2.7	6/10	20.6 ± 1.42	2.8 ± 1.7
	AIS-III (> 40°)	28	14.1 ± 2.4	11/17	20.2 ± 1.36	2.8 ± 1.8

*AIS* Adolescent idiopathic scoliosis, *SD* Standard Deviation, *N* Number

In the validation group, there are four groups, No-AIS group, AIS-I group, AIS- II group, and AIS-III group. The gender ratios were similar in each group, and there were no significant differences in age, BMI, and Risser in different groups (Table 1).

### Protein array detection in AIS

**Fig. 2:**
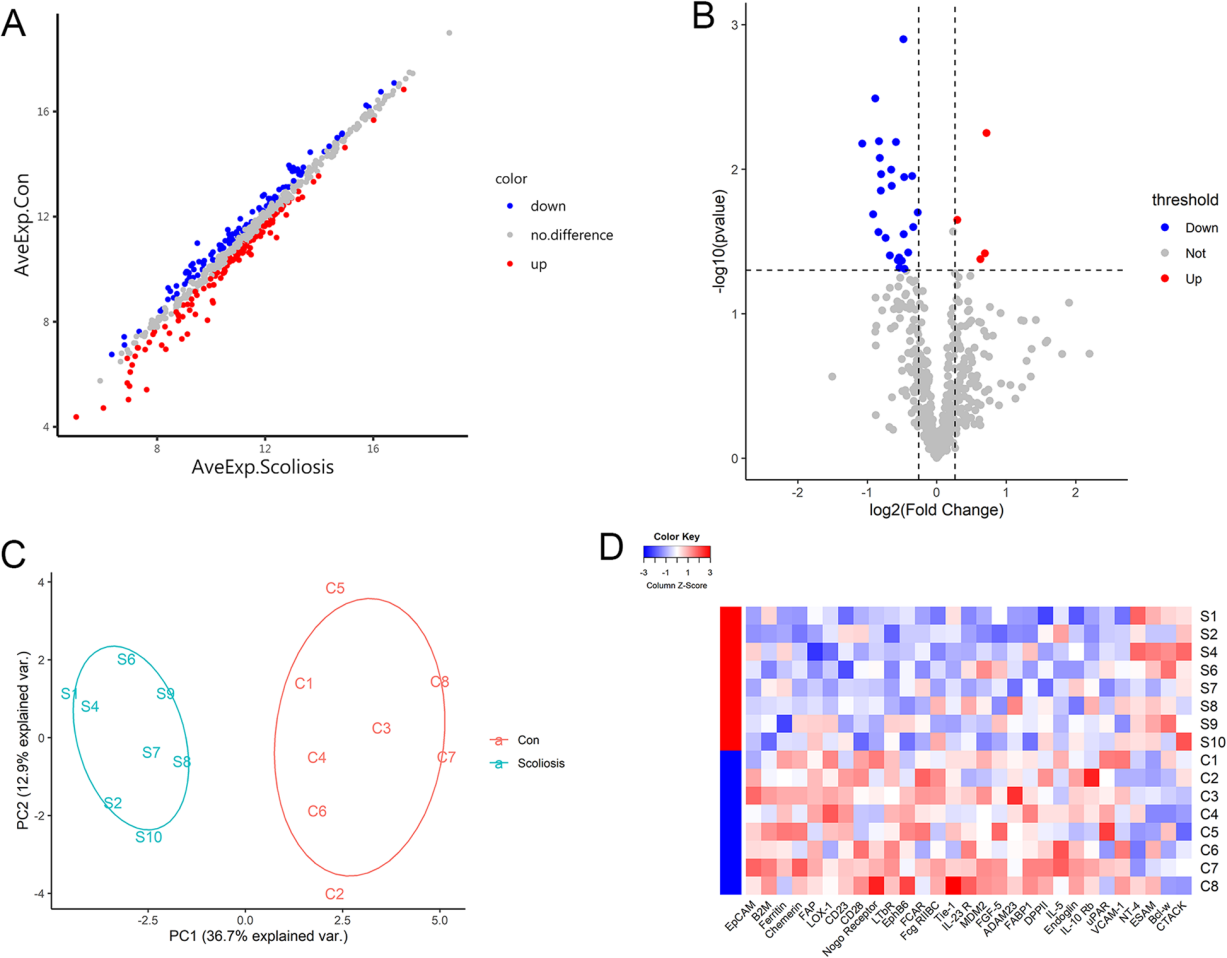
Protein expression analysis between No-AIS group and the AIS group in biomarker screening group. **A** Scatter plot showing the distribution in two groups of patients based on the average expression of proteins (AveExp). Red represents upregulation, blue represents downregulation, and gray represents no difference. **B** Volcano plot visualizing the genes separated according to their LogFC (x-axis, FC: fold change) and significance (y-axis: -log10 P. Val) in No-AIS group and the AIS group. **C** PCA was conducted on differentially expressed proteins between the two groups. The first two principal components are plotted to show the difference between the groups (control group in red and experimental group in green). **D** Microarray heat map generated by hierarchical clustering of differentially expressed proteins between groups. High signal intensities are red, and low signal intensities are blue

Protein array detection was used to identify differentially expressed proteins. The expression of 640 proteins was profiled in a cohort of 16 plasma specimens in the biomarker screening group. Among the proteins profiled, the expressions of 126 proteins were grown, and expression of 106 proteins was reduced in the AIS (fold change over 1.2 or less than 0.83) (Fig. 2A). Then the volcano plot shows there were 29 differentially expressed proteins based on that *P*- value < 0.05 (Fig. 2B). As compared with the No-AIS group, there were 4 unregulated and 25 down-regulated proteins.

In order to verify that the differential protein was sufficiently representative between No-AIS group and the AIS group, we next proceeded with principal component analysis (PCA). PCA is a common method for analyzing large datasets containing a high number of dimensions per observation, and many researches use the first two principal components to plot the data in two dimensions and to recognize clusters of closely related data points. The result of PCA showed the first two principal components can clearly separate the 16 samples into two clusters (Fig. 2C). Further analysis of differentially expressed proteins by hierarchical clustering showed great differences between No-AIS group and the AIS group (Fig. 2D).

### Top 10 differentially expressed proteins from GeneSet enrichment analysis and ROC curve

**Fig. 3:**
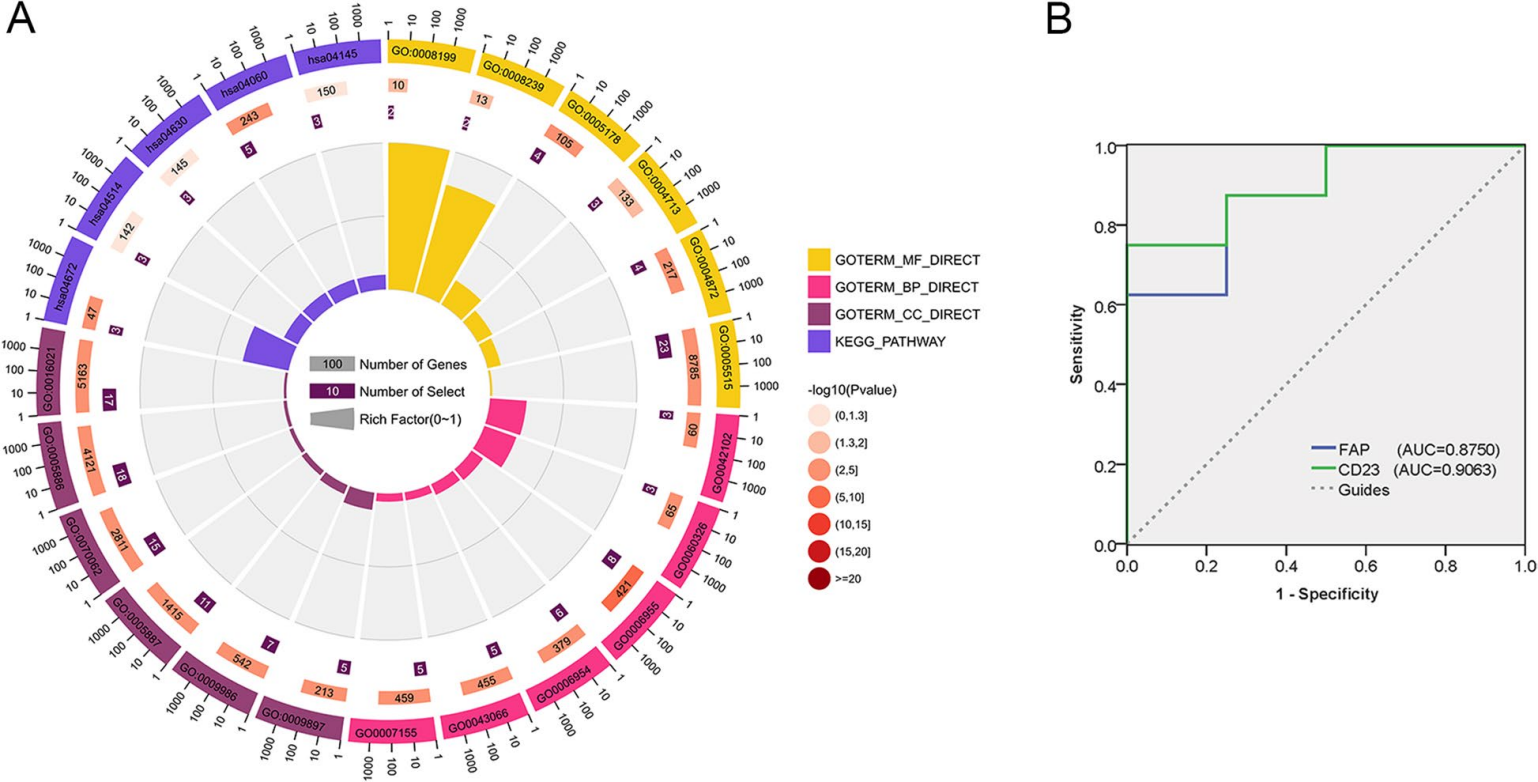
Bioinformatic analysis of gene ontology and KEGG from above differentially expressed proteins: **A** The top 6 cellular component in brown; the top 6 molecular function in yellow; the top 6 biological process in pink; the top 5 KEGG pathway enrichments in purple. **B** Receiver operating curve (ROC) analysis of CD23 and FAP. Using Fisher’s accurate test, *p* < 0.05 was considered statistically significant

To elucidate the roles of these differentially expressed proteins in AIS, GO enrichment analysis was conducted by using “clusterProfiler” package in R with a threshold of *p* less than 0.05. GO analysis contains following three functional parts: cellular components, biological processes, and molecular functions (Fig. 3A, Table S2). The results indicate that differentially expressed proteins were largely enriched in plasma membrane, extracellular exosome, integral component of plasma membrane, cell surface, external side of plasma membrane, and integral component of membrane, in the Cellular Component group; immune response, inflammatory response, positive regulation of T cell proliferation, negative regulation of apoptotic process, cell chemotaxis, and cell adhesion were listed in the Biological Processes; and differentially expressed proteins were focus on integrin binding, protein binding, receptor activity, ferric iron binding, protein tyrosine kinase activity, and dipeptidyl-peptidase activity, in the Molecular Functions group. KEGG analysis was used to acquire further biological functions in AIS through differentially expressed proteins [7, 8]. The top 5 KEGG results were listed (Table S3): Cytokine-cytokine receptor interaction, intestinal immune network for IgA production, cell adhesion molecules, Jak-STAT signaling pathway and phagosome.

The differentiating performance of these proteins is further highlighted in Fig. 3B and Figure S1, using ROC. Top 10 differentially expressed proteins were CD23, FAP, CTACK, LOX1, B2M, FcgRIIBC, IL23R, ESAM, Bclw, and DPPII (AUCs were 0.906, 0.875, 0.874, 0.859, 0.844, 0.844, 0.797, 0.781, 0.766, and 0.766, respectively).

### Correlation among demographic data and differentially expressed protein

**Fig. 4:**
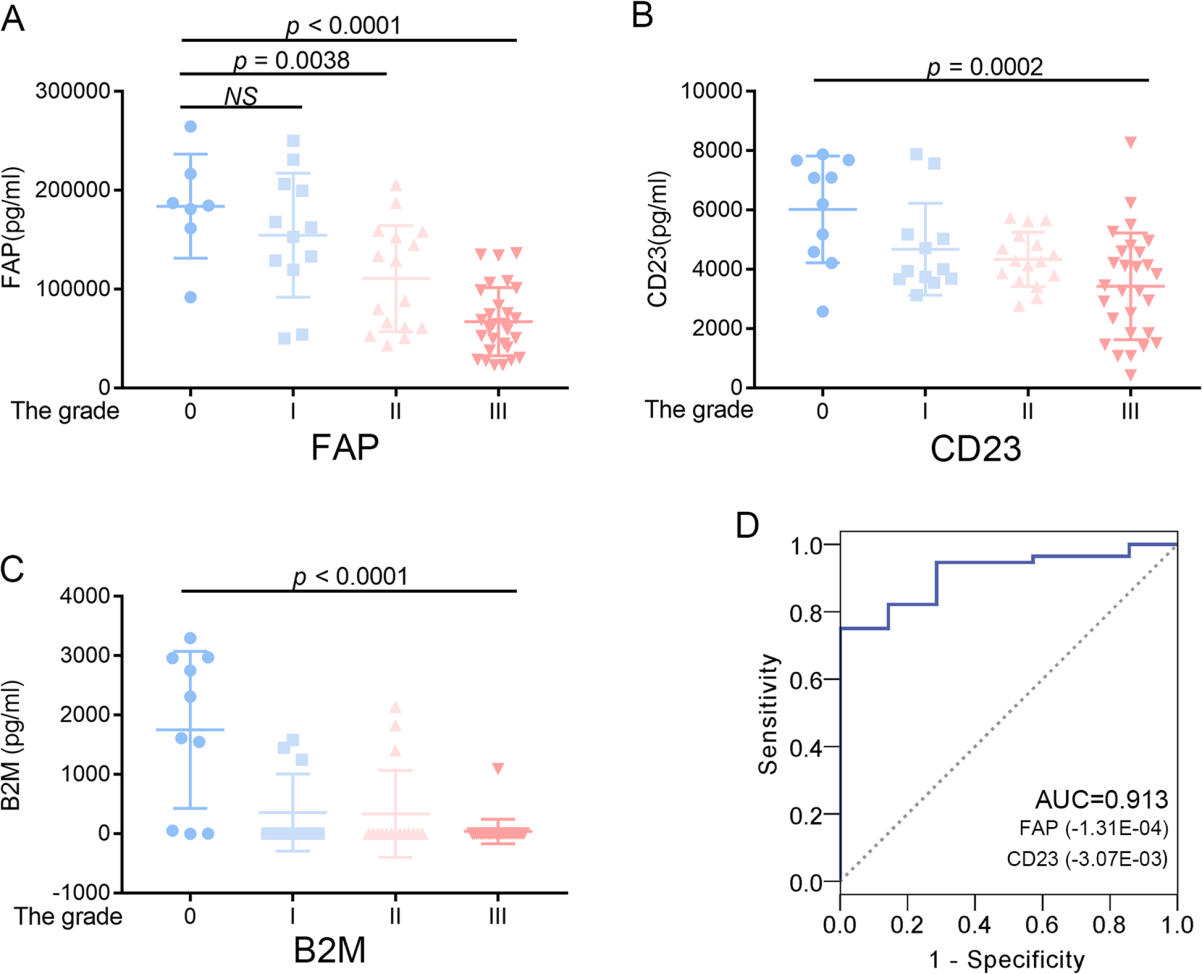
Cytokine expression and the correlation analysis in the validation group. **A** The expression of FAP were detected by ELISA. **B** The expression of CD23 and the correlation analysis. **C** The expression of B2M and the correlation analysis. **D** ROC analysis of predicted value, derived by FAP and CD23

**Fig. 5:**
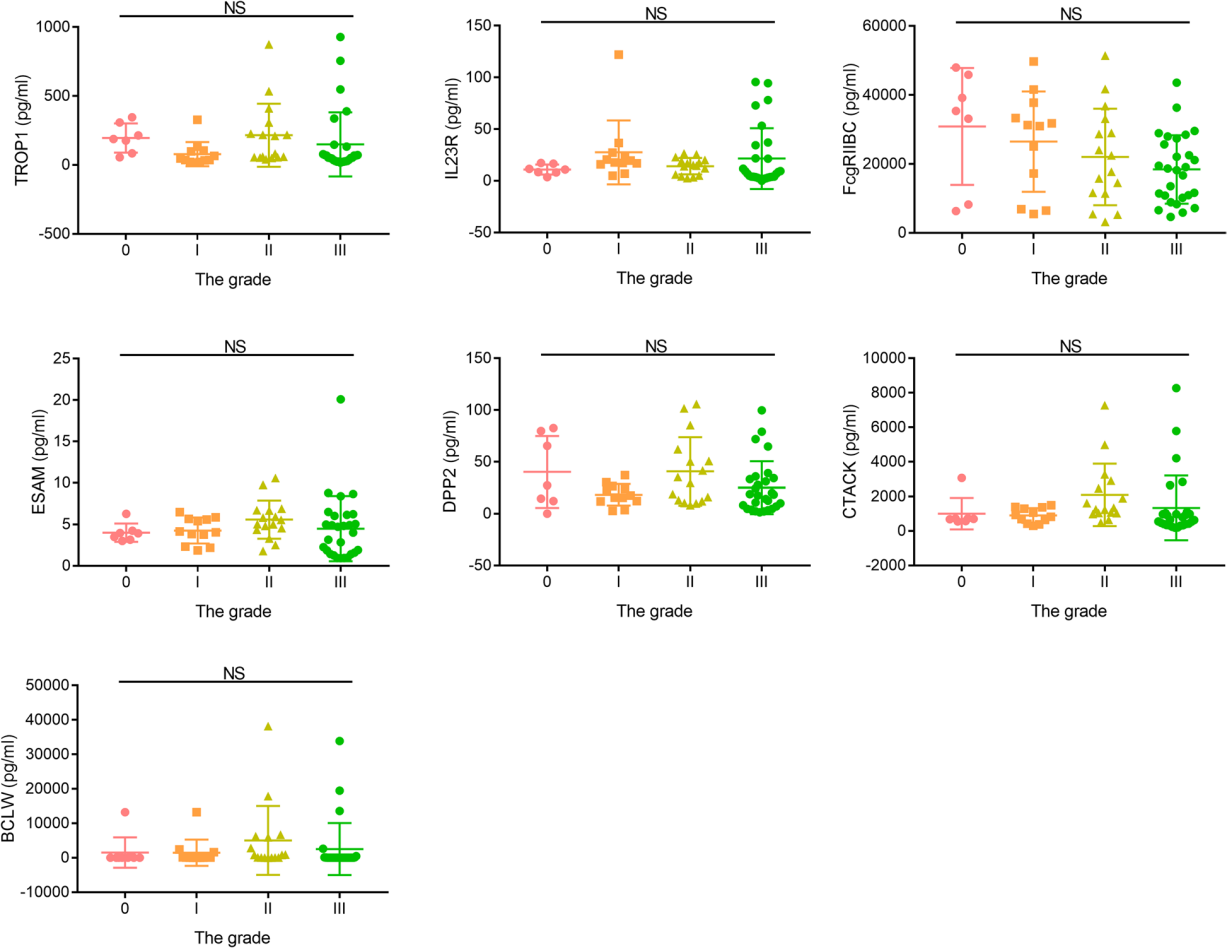
The expression of other seven proteins were detected by ELISA

In the validation group, we re-recruited 66 volunteers at different AIS degree, to verify the relationship between top 10 differentially expressed proteins and AIS. The ELISA results indicated that the expression of FAP, CD23 and B2M decreased as the degree of AIS increased (FAP, *p* < 0.0001, Fig. 4A; CD23, *p* = 0.0002, Fig. 4B; B2M, *p* < 0.0001, Fig. 4C). Meanwhile, there were no significant difference for other seven proteins in the validation group, as showed in Fig. 5.

In order to analyze the correlation between each pair of demographic parameters and differentially expressed proteins, a correlation coefficient test was used in the validation group. In the demographic data, we noticed the correlations of Age with B2M (r = -0.27; *p* < 0.05) and CD23 (r = -0.34; *p* < 0.01). In the three differentially expressed proteins, great correlations were noticed between B2M and FAP (*r* = 0.36; *p* < 0.01), B2M and CD23 (r = 0.44; *p* < 0.01), CD23 and FAP (r = 0.51; *p* < 0.01) (Table 2).

**Table 2:** Correlations among demographic data and differentially expressed protein

	**Age**	**Gender**	**B2M**	**FAP**	**CD23**
Age	1				
Gender	-0.01	1			
B2M	-0.27*	-0.02	1		
FAP	-0.16	0.25	0.36**	1	
CD23	-0.34**	0.06	0.44**	0.51**	1

*B2M* Beta-2-Microglobulin, *FAP* Fibroblast Activation Protein Alpha, CD23 Fc Epsilon Receptor II

^*^p < 0.05

^**^p < 0.01

### Multiple regression analysis between Cobb angle and potential factors.

The correlation between Cobb angle and potential factors were modeled using multiple linear regression analysis by fitting a linear equation into the data. Additionally, multiple linear regression analysis was also used to determine multicollinearity. Multicollinearity refers to a phenomenon in which one predictor variable could be predicted linearly from the others with significant precision. So in this case, small changes to the input data in the model can result in big differences, or even changing the sign of the parameter estimates.

We performed multiple regression analysis with the Cobb angle as the dependent variable and Age, FAP, CD23, and B2M as the independent variables. The results showed that FAP, CD23 are related to Cobb angle. And the Cobb angle can be reflected in the following regression equation: Cobb angle = 56.766—3.07E-03 * CD23—1.31E-04 * FAP. And two parameters Age and B2M were excluded because of multicollinearity (Table 3).

**Table 3:** Multiple regression analysis and potential factors and collinearity analysis

	**B**	**SD**	**t**	***p***	**95% Confidence Interval**		**Multicollinearity Statistics**	
					**Lower**	**Upper**	**Tolerance**	**VIF**
Constant	56.766	6.221	9.125	0.000	44.323	69.209		
CD23	-3.07E-03	0.002	-1.982	0.050	-6.16E-03	2.83E-05	0.744	1.344
FAP	-1.31E-04	0.000	-3.036	0.004	-2.17E-04	-4.47E-05	0.744	1.344

*B* Regression coefficient, *SD* Standard Deviation, *VIF* Variance inflation factor

### Verification by statistical methods

The result of variance analysis indicated the great difference between models 2 and 3 of the predictive equation at *p* < 0.05, and model 3, which had reasonable R^2^ value (Table S4, S5).

The result of collinearity analysis supported the independence of factors contributing to the regression equation (Table 3).

In the residual analysis, we use a histogram and Durbine Watson statistics to describe the appropriateness of the model. The histogram displayed that the residuals of the standard regression were prone to be normally distributed from the impression (Figure S2). Additionally, the outcome of Durbine Watson statistics analysis supported the independence of the residuals (Table S5).

**Fig. 6:**
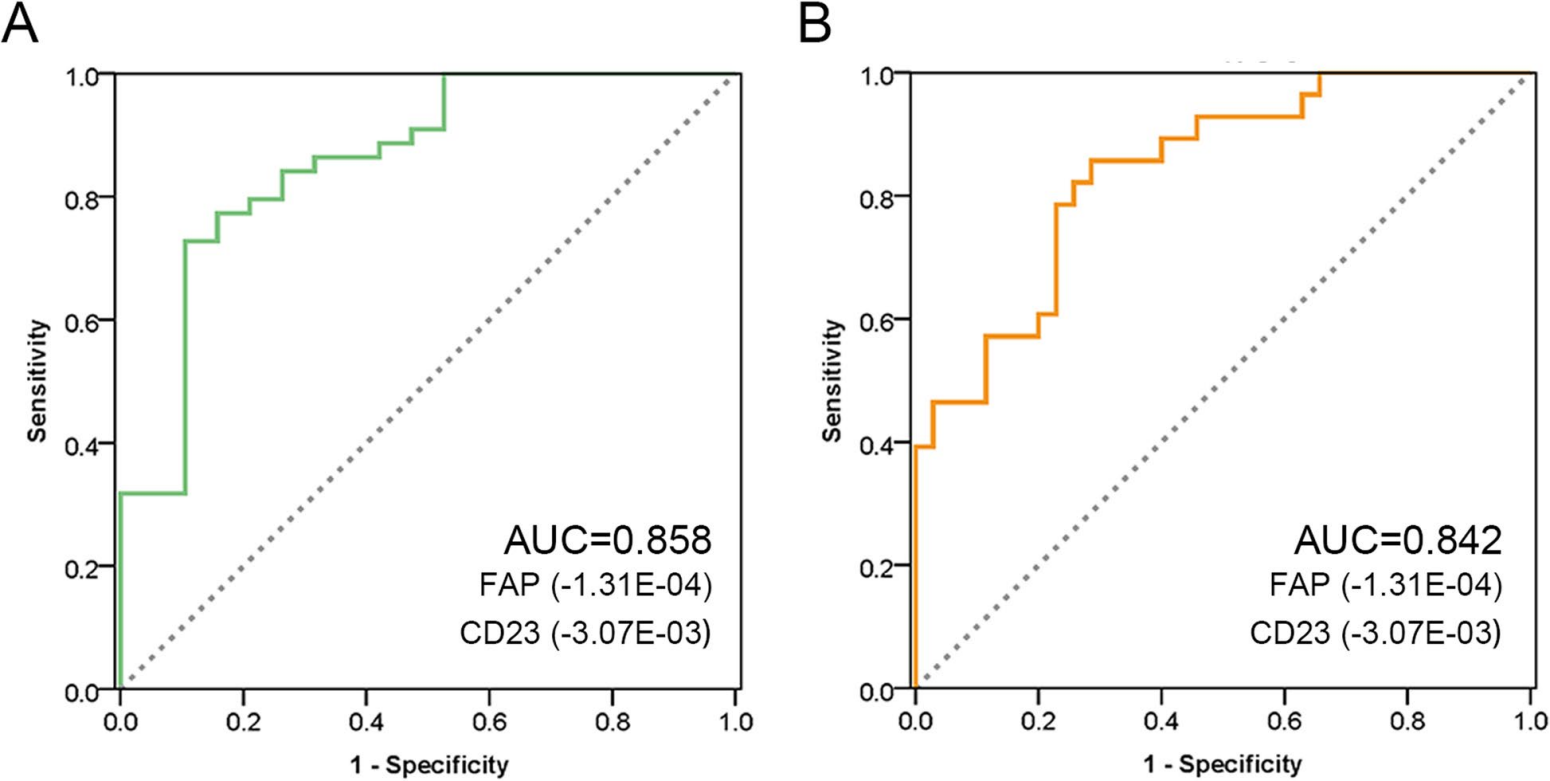
ROC analysis of predicted value, derived by FAP and CD23, including (**A**) AIS-II group and (**B**) AIS-III group

We input the verification group data into the regression equation. The predicted value, derived by FAP and CD23, can be used to distinguish No-AIS and AIS group (AUC = 0.913, Fig. 4D). And the predicted value also can be used to distinguish different AIS group (Fig. 6).

## Discussion

Evident AIS incurs high treatment costs, low quality of life, and many complications. Thus, early screening of AIS is need to avoid serious consequences. This aim of the study is to investigate some differentially expressed proteins between the No-AIS volunteers and the AIS patients to identify new biomarkers for early screening.

This study did not use the gene expression–based high-throughput screening. Despite some evidence that the condition has a genetic basis, the genetic variants that are responsible for AIS have not been identified. On the one hand, the heritability of AIS is more inclined to a complex polygenic model with considerable genetic heterogeneity; on the other hand, the formation of AIS is formed by the combination of environment and genes. Therefore, only rarely have genes been identified with putative roles in pathogenesis, despite multiple large families with an over-representation of AIS have been described [9]. Different from gene expression, proteins act as performers of function, and serum proteins serve as better screening biomarkers for AIS, such as some cancer antigens [10].

This study does not use restricted pathway proteins to avoid selection bias. Most biomarker detection methods used in the past have adopted a biased philosophy, based on the exploration of the established pathophysiological pathways associated with AIS, for instance, bone metabolism [11]. These methods, while useful, restrict the discovery of new biomarkers and related pathways. As of writing, this paper will be the first one to use an antibody-based array as a biomarker screening technique for AIS.

Using an antibody-based array, AIS patients and volunteers was interrogated for the concentrations of 640 proteins in the serum. From the preliminary screening, 10 serum proteins, from GeneSet enrichment analysis and ROC curve, were chosen for ELISA validation. From ELISA validation, the expression of FAP, CD23 and B2M decreased as the degree of AIS increased. Considering the mutual influence of various parameters, correlation analysis and Multiple regression analysis were applied for further analysis. Through the above screening and verification, we found that FAP and CD23 may serve as the biomarkers for AIS.

FAP deficiency may affect endplate cartilage to promote the development of AIS. FAP is a homodimeric integral membrane gelatinase which is thought to play a role in the control of fibroblast growth or epithelial-mesenchymal interactions during development, tissue repair, and epithelial carcinogenesis. The results of geneSet enrichment analysis showed that plasma membrane, cell surface, and integral component of membrane were underlined in the cellular component group; cell adhesion was listed in the biological processes; and integrin binding, protein binding and dipeptidyl-peptidase activity were emphasized in the molecular functions group. In the cartilage, previous studies had demonstrated that FAP^+^ fibroblasts affect cartilage damage and inflammation [12], and deficiency of FAP also affects cartilage destruction in inflammatory destructive arthritis [13]. Meanwhile, Duance et al*.* have characterized the apparent differences in scoliosis endplate cartilage [14], and Roberts et al*.* analyzed endplate cartilage to demonstrated that the proteoglycan content was considerably lower in endplate from scoliosis samples [15]. Therefore, FAP is likely to affect scoliosis by affecting the endplate cartilage, and we prove that FAP was negatively correlated with the AIS degree for the first time (Figure S3).

Immune system disorder reflected by CD23 is closely related to AIS. CD23 plays an important role in follow terms: plasma membrane, extracellular exosome, integral component of plasma membrane, external side of plasma membrane, and integral component of membrane were underlined in the cellular component group, in the geneSet enrichment analysis; integrin binding and protein binding were emphasized in the molecular functions group. CD23 has a critical role in immune cells B cells growth and differentiation, and the regulation of IgE production. Previous studies have described the presence of fibrosis in paraspinal muscle in AIS [16, 17], and the immune system is one of the primary players in the muscle remodeling through inflammation [18]. Araya et al*.* and Freeman et al*.* found that patients with hyperimmunoglobulin E syndrome had idiopathic scoliosis, because the immune deficiencies could lead to abnormalities in the bones and muscles [19, 20]. Thus, CD23 may be related to the scoliosis through immune cell infiltration in bones and muscle.

The reduction of serum B2M in patients with severe AIS may be related to the renal dysfunction associated with AIS. B2M is a serum protein found in association with the major histocompatibility complex (MHC) class I heavy chain on the surface of nearly all nucleated cells. The protein has a predominantly beta-pleated sheet structure that can form amyloid fibrils in some pathological conditions. Gao et al*.* found that the expression of B2M in the excreted urine was increased in the scoliosis children [21], and Suzuki et al*.* also found that increased excretion of B2M through urine in idiopathic scoliosis patients with urinary tract obstruction [22]. Correspondingly, we found that the serum B2M in patients with severe AIS was reduced detected by protein array and ELSIA, which may be caused by excessive excretion through urine (Fig. 4C). In addition, Yuki et al*.* found that B2M was linked to cartilage development from pluripotent stem cell [23]. The function of B2M and FAP may be similar and related (Table 2, r = 0.36; *p* < 0.01), but the result of multiple regression analysis points to FAP.

Several improvements could be made to this study. A larger sample size and inclusion of additional ethnic groups would help provide more power to validate. In addition, mechanisms are required to explore the cellular origins of the identified biomarkers and to elucidate the role of each in disease pathogenesis.

## Conclusion

A targeted antibody-based array is expected to reveal a serum protein signature as biomarker for AIS, and this paper is the first to use the unbiased planar array screening of 640 proteins as a biomarker screening technique for AIS. Interestingly, we found that serum protein FAP and CD23 are intimately related to AIS, suggesting FAP and CD23 are expected to serve as the serum biomarkers, which significantly facilitate frequent longitudinal monitoring as to keep track of disease progression and tailor treatment accordingly.

## Methods

### Patient samples

As for the biomarker screening group, from April 2020 to August 2020, 16 volunteers were recruited in our study, including 8 volunteers and 8 adolescent idiopathic scoliosis patients in the Second Affiliated Hospital of Zhejiang University, and the Second Affiliated Hospital of Wenzhou medical university.

As for the validation group, from October 2020 to February 2021, a total of 80 volunteers were enrolled in the Second Affiliated Hospital of Zhejiang University, and the Second Affiliated Hospital of Wenzhou medical university. However, 8 volunteers left with only image data, and 6 blood samples of volunteers were unqualified and could not be tested by ELISA.

This study was approved by the local ethics committee. Written informed consent was obtained from every volunteer before collecting patient information for analysis, for example, coronary radiographs, and serum samples. The grade of scoliosis is classified by the Cobb angle of main curve in the coronal radiographs.

### Protein array assay

In order to avoid degradation of serum proteins, we store and transport samples at -80 °C, and remove low-quality samples in time when carrying out pre-experiments. In terms of the initial screening of biomarkers, the supernatant was analyzed using a glass-based and sandwich-based antibody microarray to measure 640 human proteins quantitatively (QAH-CAA-640, RayBiotech, Peachtree Corners, Georgia, USA). In terms of the next round of screening, we constructed a custom glass-based antibody array for targeting proteins (RayBiotech, Peachtree Corners, Georgia, USA). Each protein was analysed in quadruplicate per array.

### Gene ontology and pathway enrichment analyses

To gain a deeper understanding of the functions of the differentially expressed proteins, Gene ontology (GO) enrichment and Kyoto Encyclopedia of Genes and Genomes (KEGG) pathway analyses were conducted to determine the functions in which the differentially expressed proteins participated [7, 8]. Functional enrichment analyses were based on Fisher's exact test in the clusterProfiler package of R/Bioconductor, and the threshold values were set as count ≥ 3 and *P*-value < 0.05.

### Enzyme-Linked immunosorbent assay (ELISA)

From above analysis in the biomarker screening group, we got top 10 biomarkers. Then the concentrations of these proteins were analyzed using enzyme linked immunosorbent assay (ELISA) kits.

### Statistical analysis

All statistical analyses were performed with a SPSS version 19.0 (SPSS Inc, Chicago, IL, USA) and GraphPad Prism software Version 5 (GraphPad Software, Inc, 220 San Diego, CA), and descriptive parameters were in form of mean ± standard deviation. Correlations parameters were analyzed by ANOVA statistical analyses. A probability (*p*) value < 0.05 was considered statistically significant.

### Supplementary Information

**Additional file 1: Figure S1. **Receiver operating curve (ROC) analysis of CTACK, LOX1, B2M, FcgRIIBC, IL23R, ESAM, Bclw, and DPPII (AUCs were 0.906, 0.875, 0.874, 0.859, 0.844, 0.844, 0.797, 0.781, 0.766, and 0.766, respectively).** Figure S2.** Histogram of residual analysis. Std, standard deviation. **Figure S3.** Histochemical results of paravertebral tissue showed that the expression of FAP and CD23 in AIS-III was lower than that in the control group.

**Additional file 2: Table S1. **Descriptive statistics are shown for the SF-36 questionnaires. **Table S2.** The top 6 enriched gene ontology terms of the differentially expressed proteins. **Table S3.** The top 5 enriched KEGG pathways of the differentially expressed proteins. **Table S4.** Multiple Regression Analysis and Potential Factors and Collinearity Analysis. **Table S5.** Residual Analysis.

**Additional file 3.** Appendix Supplementary methods.

## Data Availability

All data used during the study are available in Zenodo (https://doi.org/10.5281/zenodo.7698680).

## Abbreviations

AIS: Adolescent idiopathic scoliosis
FBT: Forward bending test
ATR: Angle of trunk rotation
ELISA: Enzyme-Linked immunosorbent assay
PCA: Principal component analysis
GO: Gene ontology
KEGG: Kyoto Encyclopedia of Genes and Genomes
ROC: Receiver operating curve
CD: Cluster of differentiation
FAP: Fetoacinar pancreatic protein
CTACK: Cutaneous T cell-attracting chemokine
LOX: Lectin-like oxidized LDL receptor
IL: Interleukin
ESAM: Endothelial cell adhesion molecule
FcgRIIB: Fc Gamma Receptor IIb
Bcl: B cell leukemia
DPP: Dipeptidyl peptidase
