# Supplementary material for: A targeted antibody-based array reveals a serum protein signature as biomarker for adolescent idiopathic scoliosis patients
Source: BMC Genomics. 2023 Sep 4;24:522. doi: 10.1186/s12864-023-09624-7 (PMC10478410; doi:10.1186/s12864-023-09624-7)
Supplement: Supplementary file 1 — Additional file 1: Figure S1. Receiver operating curve (ROC) analysis of CTACK, LOX1, B2M, FcgRIIBC, IL23R, ESAM, Bclw, and DPPII (AUCs were 0.906, 0.875, 0.874, 0.859, 0.844, 0.844, 0.797, 0.781, 0.766, and 0.766, respectively). Figure S2. Histogram of residual analysis. Std, standard deviation. Figure S3. Histochemical results of paravertebral tissue showed that the expression of FAP and CD23 in AIS-III was lower than that in the control group. [file 12864_2023_9624_MOESM1_ESM.docx]

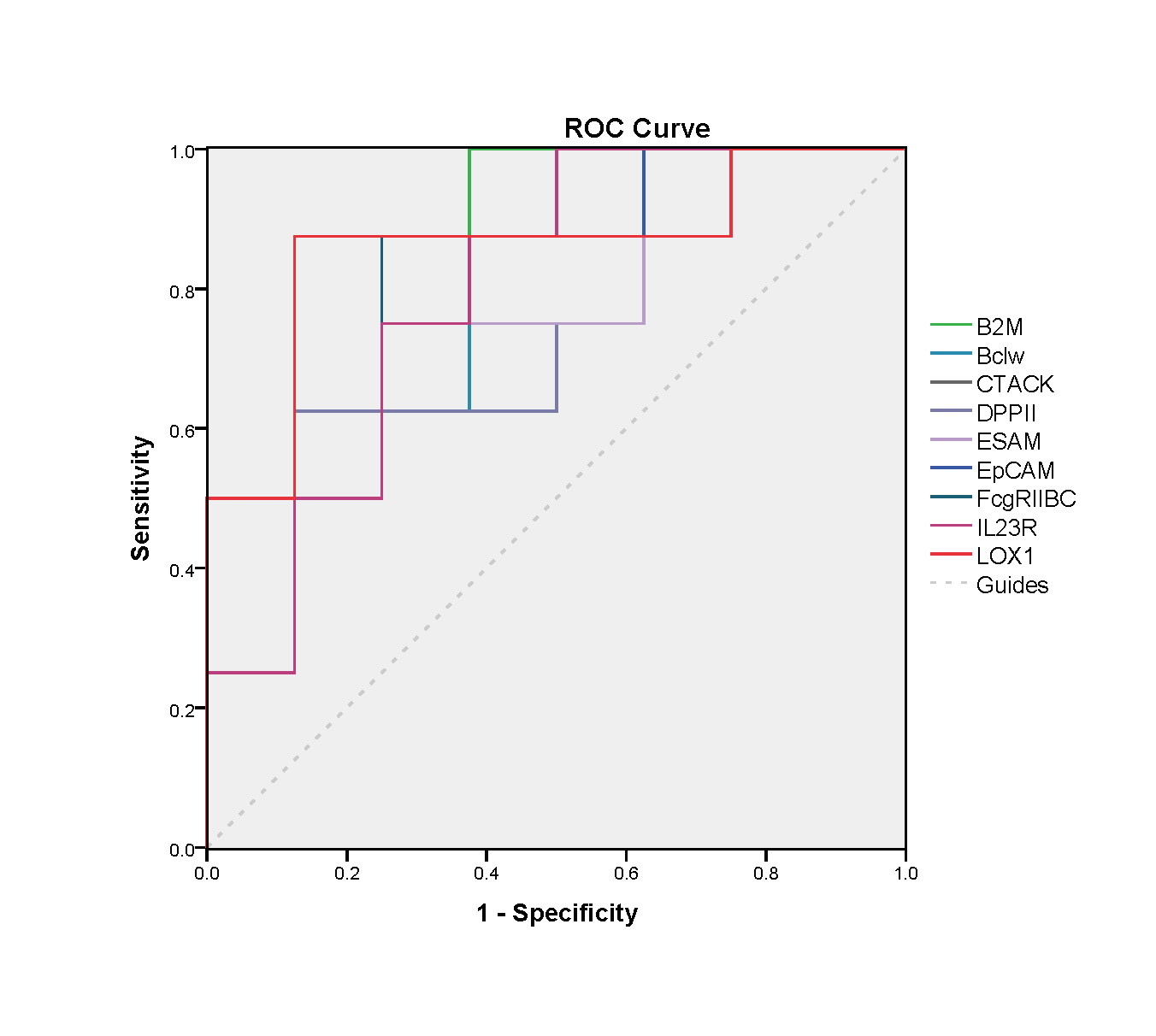


**Figure S1.** Receiver operating curve (ROC) analysis of CTACK, LOX1, B2M, FcgRIIBC, IL23R, ESAM, Bclw, and DPPII (AUCs were 0.906, 0.875, 0.874, 0.859, 0.844, 0.844, 0.797, 0.781, 0.766, and 0.766, respectively).


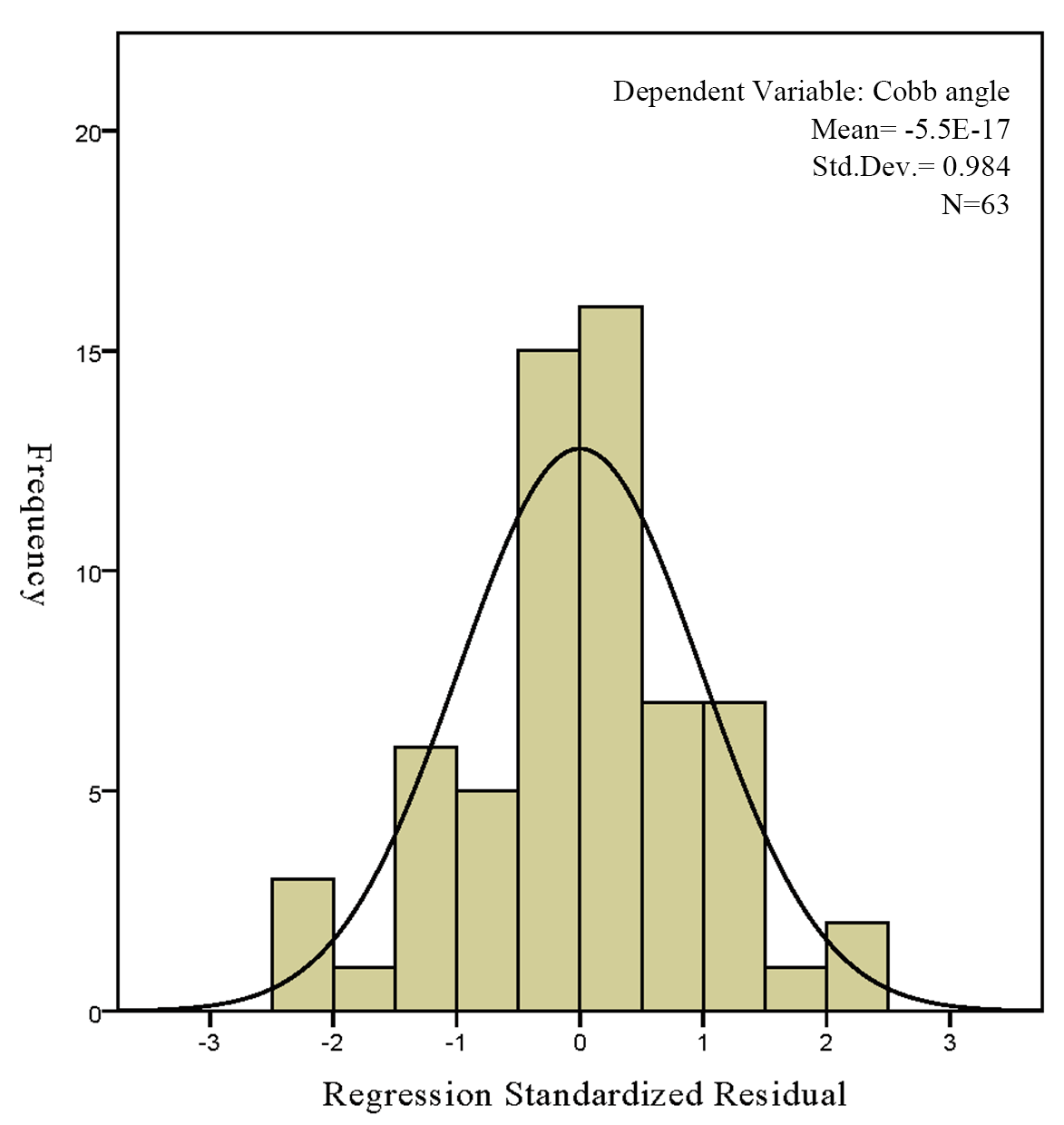


**Figure S2.** Histogram of residual analysis. Std, standard deviation.


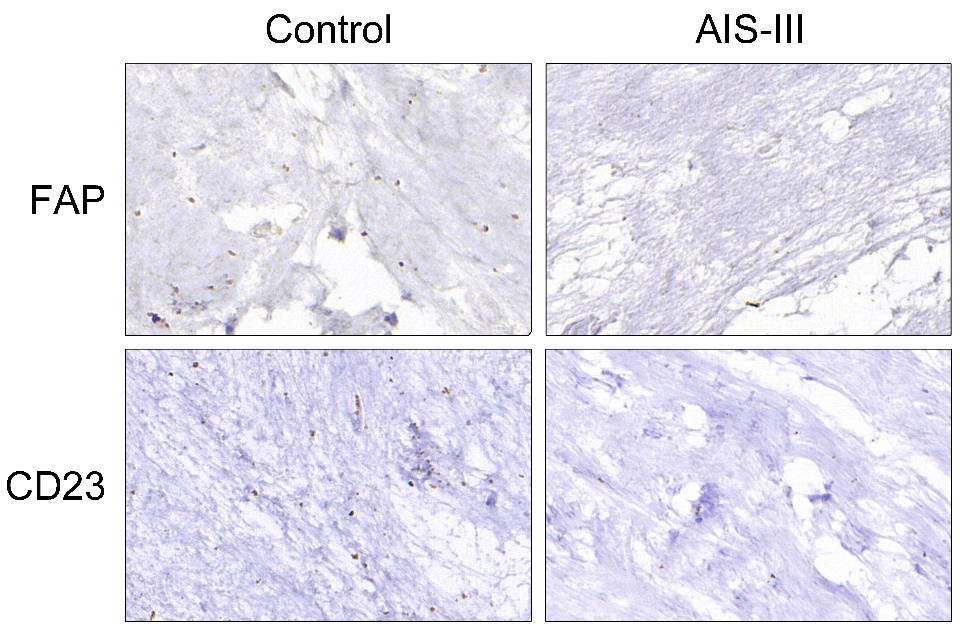


**Figure S3.** Histochemical results of paravertebral tissue showed that the expression of FAP and CD23 in AIS-III was lower than that in the control group.
