# Supplementary material for: A targeted antibody-based array reveals a serum protein signature as biomarker for adolescent idiopathic scoliosis patients
Source: BMC Genomics. 2023 Sep 4;24:522. doi: 10.1186/s12864-023-09624-7 (PMC10478410; doi:10.1186/s12864-023-09624-7)
Supplement: Supplementary file 2 — Additional file 2: Table S1. Descriptive statistics are shown for the SF-36 questionnaires. Table S2. The top 6 enriched gene ontology terms of the differentially expressed proteins. Table S3. The top 5 enriched KEGG pathways of the differentially expressed proteins. Table S4. Multiple Regression Analysis and Potential Factors and Collinearity Analysis. Table S5. Residual Analysis. [file 12864_2023_9624_MOESM2_ESM.docx]

| **Table S1: Descriptive statistics are shown for the SF-36 questionnaires** | | | | |
| --- | --- | --- | --- | --- |
|  | No-AIS (0°-10°) | AIS-I (10°-20°) | AIS-II (20°-40°) | AIS-III (>40°) |
| Physical function | 94±5.5 | 90±12.2 | 65±8.7 | 62±11.0 |
| Role-physical | 90±13.7 | 85±13.7 | 60±13.7 | 55±11.2 |
| Bodily pain | 94±8.8 | 90±8.8 | 85±9.3 | 79±17.6 |
| General health | 88±9.7 | 84±9.3 | 82±11.5 | 76±7.4 |
| Validity | 83±16.0 | 79±12.9 | 74±19.5 | 71±5.5 |
| Social function | 93±6.6 | 90±16.1 | 85±16.1 | 83±11.2 |
| Role-emotional | 93±14.8 | 87±18.1 | 80±18.1 | 80±18.1 |
| Mental health | 90±9.2 | 90±9.6 | 82±15.9 | 76±11.3 |
| AIS, Adolescent idiopathic scoliosis | | | | |

| Table S2: The top 6 enriched gene ontology terms of the differentially expressed proteins. | | | |
| --- | --- | --- | --- |
| **Cellular component group** | | | |
| **Term** | **Description** | **P.Val** | **Gene** |
| GO:0005886 | plasma membrane | 0.0000 | EPHB6, Nogo Receptor, VCAM1, IL-10 Rb, IL23R, uPAR, FCAR, CD23, FAP, EPCAM, ADAM23, MDM2, CD28, LOX-1, ESAM, LTBR, Fcg RIIBC, B2M |
| GO:0070062 | extracellular exosome | 0.0000 | Nogo Receptor, VCAM1, IL-10 Rb, Chemerin, uPAR, FABP1, CD23, DPPII, EPCAM,  LOX-1, ESAM, B2M, Bcl-w, Ferritin |
| GO:0005887 | integral component of plasma membrane | 0.0000 | EPHB6, Nogo Receptor, CD23, EPCAM, Tie-1, ADAM23, uPAR, CD28, LOX-1, LTBR, FCAR |
| GO:0009986 | cell surface | 0.0002 | EPHB6, Nogo Receptor, VCAM1, FAP, EPCAM, CD28, Endoglin |
| GO:0009897 | external side of plasma membrane | 0.0003 | CD23, VCAM1, CD28, B2M, Endoglin |
| GO:0016021 | integral component of membrane | 0.0021 | EPHB6, VCAM1, IL-10 Rb, Tie-1, IL23R, uPAR, FCAR, CD23, FAP, EPCAM, Ferritin, ADAM23, LOX-1, ESAM, Fcg RIIBC, Bcl-w, Endoglin |
| **Biological processes group** | | | |
| **Term** | **Description** | **P.Val** | **Gene** |
| GO0006955 | immune response | 0.0000 | IL-5, IL-10 Rb, Ferritin, LTBR, Fcg RIIBC, B2M, CTACK, FCAR |
| GO0006954 | inflammatory response | 0.0004 | IL-5, IL-10 Rb, IL23R, Chemerin, LOX-1, LTBR |
| GO0042102 | positive regulation of T cell proliferation | 0.0048 | VCAM1, IL23R, CD28 |
| GO0060326 | cell chemotaxis | 0.0056 | VCAM1, CTACK, Endoglin |
| GO0043066 | negative regulation of apoptotic process | 0.0074 | FABP1, EPCAM, uPAR, MDM2, Bcl-w |
| GO0007155 | cell adhesion | 0.0076 | VCAM1, FAP, ADAM23, LOX-1, Endoglin |
| **Molecular function group** | | | |
| **Term** | **Description** | **P.Val** | **Gene** |
| GO:0005178 | integrin binding | 0.0007 | CD23, VCAM1, FAP, ADAM23 |
| GO:0005515 | protein binding | 0.0036 | Nogo Receptor, IL-10 Rb, Tie-1, IL23R, Chemerin, uPAR, NT-4, FABP1, CD23, IL-5, FAP, EPCAM, ADAM23, MDM2, CD28, LOX-1, LTBR, Fcg RIIBC, B2M, Bcl-w, Endoglin, Ferritin |
| GO:0004872 | receptor activity | 0.0054 | EPHB6, Nogo Receptor, IL-10 Rb, uPAR |
| GO:0008199 | ferric iron binding | 0.0165 | FTH1,  FTL |
| GO:0004713 | protein tyrosine kinase activity | 0.0204 | FGF5, IL-5, Tie-1 |
| GO:0008239 | dipeptidyl-peptidase activity | 0.0214 | DPPII, FAP |

| Table S3: The top 5 enriched KEGG pathways of the differentially expressed proteins. | | | |
| --- | --- | --- | --- |
| **Term** | **Description** | **P.Val** | **Gene** |
| hsa04060 | Cytokine-cytokine receptor interaction | 0.0057 | IL-5, IL-10 Rb, IL23R, LTBR, CTACK |
| hsa04672 | Intestinal immune network for IgA production | 0.0088 | IL-5, CD28, LTBR |
| hsa04514 | Cell adhesion molecules (CAMs) | 0.0688 | VCAM1, CD28, ESAM |
| hsa04630 | Jak-STAT signaling pathway | 0.0714 | IL-5, IL-10 Rb, IL23R |
| hsa04145 | Phagosome | 0.0757 | LOX-1, Fcg RIIBC, FCAR |
|  |  |  |  |

| Table S4: Multiple Regression Analysis and Potential Factors and Collinearity Analysis. | | | | | | |
| --- | --- | --- | --- | --- | --- | --- |
| Model |  | Sum of Squares | DF | Mean Square | F | *p* |
| 1 | Regression | 9290.81 | 4.00 | 2322.70 | 6.626 | 0.000 |
|  | Residual | 20330.37 | 58.00 | 350.52 |  |  |
|  | Total | 29621.18 | 62.00 |  |  |  |
| 2 | Regression | 9285.85 | 3.00 | 3095.28 | 8.981 | 0.000 |
|  | Residual | 20335.33 | 59.00 | 344.67 |  |  |
|  | Total | 29621.18 | 62.00 |  |  |  |
| 3 | Regression | 8920.90 | 2.00 | 4460.45 | 12.929 | 0.000 |
|  | Residual | 20700.28 | 60.00 | 345.01 |  |  |
|  | Total | 29621.18 | 62.00 |  |  |  |
| DF, degrees of freedom.   1. Predictors: (Constant), FAP, CD23, B2M, Age 2. Predictors: (Constant), FAP, CD23, B2M 3. Predictors: (Constant), FAP, CD23 | | | | | | |

| Table S5: Residual Analysis. | | | | |
| --- | --- | --- | --- | --- |
| **Model** | **R** | **R Square** | **Standard Error** | **Durbin-Watson** |
| 1 | 0.560 | 0.314 | 0.266 |  |
| 2 | 0.560 | 0.313 | 0.279 |  |
| 3 | 0.549 | 0.301 | 0.278 | 1.399 |
| DF, degrees of freedom.  1. Predictors: (Constant), FAP, CD23, B2M, Age  2. Predictors: (Constant), FAP, CD23, B2M  3. Predictors: (Constant), FAP, CD23 | | | | |
