# Supplementary material for: A targeted antibody-based array reveals a serum protein signature as biomarker for adolescent idiopathic scoliosis patients
Source: BMC Genomics. 2023 Sep 4;24:522. doi: 10.1186/s12864-023-09624-7 (PMC10478410; doi:10.1186/s12864-023-09624-7)
Supplement: Supplementary file 3 — Additional file 3. Appendix Supplementary methods. [file 12864_2023_9624_MOESM3_ESM.docx]

**Appendix**

**Supplementary methods**

***Data preparation***

In brief, 100µL of twofold diluted culture supernatant was added to each well, incubated overnight at 4°C and extensively washed. A biotin-labelled detection antibody was added for 2hours, and then AlexaFluor 555-conjugated streptavidin was applied for 1hour at room temperature. The slides were analysed with 532nm excitation and 635nm emission using an InnoScan 300 Scanner (Innopsys, Carbonne, France).

Raw data from the array scanner were provided as image files (.tif files) and spot intensities (tab-delimited.txt file) through Mapix 7.3.1 Software. Data visualisation was performed using Q-Analyzer Software (RayBiotech, Peachtree Corners, Georgi, USA). Median pixel intensities of the local background were subtracted from the median pixel intensities of individual array spots. The average spot intensity across quadruplicate spots was calculated. Interslide and intraslide signals were normalized using positive control spots. Spots with a signal intensity <5% above background were defined as non- detectable.

***Differentially expressed genes screening***

Interslide normalization was calculated using the third diluted standard (‘Standard 3’) and internal controls. For protein quantification, standard curves were generated using purified proteins representing the proteins-of-interest. Proteins were excluded from analyses if they had concentrations below the detection limit for over 50% of samples, resulting in a total of 640 quantifiable proteins. All the raw data were log2-transformed for statistical analysis, including supervised and bioinformatics analyses.

***Principal component analysis and Hierarchical clustering analysis***

Principal component analysis (PCA) was performed using the “prcomp” command and visualized using the “ggfortify” package in R/ Github.

Hierarchical clustering analysis was performed based on the expression values to generate an overview of the protein expression profile between the AIS and NO-AIS groups. We visualized the expression profile via heatmaps showing hierarchical clustering by Euclidian distance and complete linkage using the heatmap.2 function in the “gplots” package for statistical computing.
